# Impact of organizational characteristics on employees’ entrepreneurial orientation with mediating role of knowledge process capabilities and moderating role of psychological factors in the era of COVID-19

**DOI:** 10.3389/fpsyg.2022.799149

**Published:** 2022-12-14

**Authors:** Muhammad Farhan Basheer, Saeed Ahmad Sabir, Rabeeya Raoof, Waseem Ul Hameed, Saida Jabeen

**Affiliations:** ^1^Lahore Business School, The University of Lahore, Lahore, Pakistan; ^2^Hailey College of Commerce, University of the Punjab, Lahore, Pakistan; ^3^Institute of Business, Management and Administrative Sciences, The Islamia University of Bahawalpur, Bahawalpur, Pakistan; ^4^Lahore Business School, The University of Lahore, Islamabad, Pakistan

**Keywords:** knowledge management, entrepreneurial orientation, organizational characteristics, resource-based view, psychological factors, COVID-19

## Abstract

**Purpose:**

The study aims to investigate the impact of organizational characteristics and knowledge process capabilities on the entrepreneurial orientation among the manufacturing industry employees in the Punjab province of Pakistan. Additionally, this study has examined the mediating role of knowledge process capabilities in the relationship between organizational characteristics and entrepreneurial orientation among those employees and the moderating effect of psychological factors on the relationship between organizational characteristics and entrepreneurial orientation.

**Design, methodology, and approach:**

The study has employed the survey-based methodology and data are collected with the aid of self-administered questionnaires. This study utilized the partial least squares structural equation modeling (PLS-SEM) to establish the validity and reliability of the measurement model and test the relationships. The response rate of the current study is 64.66%.

**Findings:**

The study findings have shown mixed results as one of the organizational characteristics, namely, resource and time availability is an insignificant determinate of entrepreneurial orientation among the manufacturing industry employees in Punjab province of Pakistan. Whereas management support, rewards, work discretion, and knowledge process capabilities appear as significant determinates of employees’ entrepreneurial orientation. The results indicated that knowledge process capabilities have a mediating role in the relationship between organizational characteristics and employees’ entrepreneurial orientation. Moreover, psychological factors, namely, propensity to take risk and locus of control have a significant moderating role on the relationship of management support, rewards, and work discretion with employees’ entrepreneurial orientation.

**Practical implications:**

The empirical insights on the study are valuable for policymakers and managers in manufacturing sectors of developing countries, such as Pakistan, to enrich their work performance through the understanding impact of organizational characteristics and knowledge process capabilities on the entrepreneurial orientation with moderating role of psychological factors.

**Originality and value:**

Studies on the mediating impact of knowledge process capabilities on the linkage between organizational characteristics and entrepreneurial orientation with the moderating role of psychological factors remain limited. This study is one of the earliest studies that investigate these inter-relationships.

## Introduction

The challenges faced by the organizations in manufacturing industries have become greater as a result of globalization whereby their customers have developed higher prerequisites (Mufti et al., 2020). In addition, COVID-19 coronavirus disease, 2019, caused by a new kind of virus, namely, coronavirus (SARS-CoV-2), is a serious pandemic. The first case of COVID-19 was reported in December 2019 in Wuhan, China. After that, this virus spread all around the globe rapidly (Holden et al., 2021), and approximately 5,000,000 cases were reported with around 300,000 casualties. In March 2020, World Health Organization (WHO) announced officially a state of the epidemic by involving 114 countries around the globe (Hernández-Sánchez et al., 2020). This pandemic proved a violent shock by creating a highly uncertain and unprecedented environment not only for the health of humanity but also for the economy of the globe (Gómez-Salgado et al., 2020). Already, countries were investing a large share of their budget in healthcare capacity building and economic system development particularly for educated people, but the situation remained miserable. Illustratively, COVID-19 witnessed to have an adverse influence on the physical and psychological aspects of health (Wang et al., 2020). Concerning China, 58.3% of the people are facing the psychological issues of decrease in positive feelings and life satisfaction (Shehzadi et al., 2020; Wang et al., 2020).

Hence, to survive and ultimately succeed, manufacturers need to expand their profitability by having specific end goals (Parida and Pradhan, 2016; Testaverde et al., 2017; Mufti et al., 2020). These organizations can be more aggressive by improving their manufacturing efficiency and fulfilling the ever-changing needs of their customers and employees. Additionally, on the top of proving their manufacturing capabilities, manufacturing companies can also apply excellent manufacturing practices that would improve their customization capacities (Testaverde et al., 2017; Mufti et al., 2020). In the context of Malaysia’s manufacturing industries, companies are now undergoing expansions to improve their operational execution (Kesavan, 2018). This includes reducing the life cycle of products, thus resulting in unpredictable data costs which force manufacturers to be flexible, resourceful, receptive, and inventive (Testaverde et al., 2017; Mufti et al., 2020). Prior to this, manufacturing companies only need to tackle the aspects of cost and quality; today, they need to take into account all the aspects of manufacturing while being adaptable and responsive to the present economic landscape (Testaverde et al., 2017; Mufti et al., 2020).

As such, there is a need to identify the status of Malaysian manufacturing companies and compare it to that of other companies applying best manufacturing practices. This can help the companies to identify and focus on the areas that need change. This will also prove that the companies are mindful, thus improving their execution and strength. Hence, by adopting best manufacturing practices, these companies can improve their business execution and organizational resources, thus creating new work opportunities and growing the manufacturing industry as a whole (Ali et al., 2017; Testaverde et al., 2017; Mufti et al., 2020) on the top of improving the country’s economic progress (Phang et al., 2017). The manufacturing situation does not show a good sign for the nation’s economy. Nevertheless, employees must also be willing to implement adaptable manufacturing systems *via* the adoption and usage of new technologies; by doing so, the employees will gain the basic knowledge that can facilitate their individual growth, productivity, and performance, hence ensuring long-term and sustainable organizational growth (Testaverde et al., 2017; Mufti et al., 2020). Employees’ relations in manufacturing companies are similar to open systems whereby external environmental changes can affect internal dynamics in various ways (Vanchan et al., 2018). In short, the survivability and success of manufacturing companies require both organizational and individual entrepreneurship (Fisher et al., 2020). Organizational re-developments are therefore projected to occur following the implementation of entrepreneurship skills among the employees.

This is essential considering the present-time dynamics and competitive uncertainties which force manufacturing companies to obtain the necessary resources for developing viable strategies (Gunasekaran et al., 2018; Kharub et al., 2019; Mufti et al., 2020). Yet, there is still a heated debate on whether the employees of manufacturing companies can coordinate their entrepreneurial capacities and behavior. Due to this, glaring research gaps remain in manufacturing literature, particularly because a majority of the existing studies had only focused on the organizational level rather than on a regional basis (Hernández et al., 2017). The studies also only focused on the inputs of managing directors and business owners while neglecting those of the employees (Sahasranamam and Sud, 2016). Kraus et al. (2019) asserted that entrepreneurial orientation should be captured by individual employees from the operational level. A majority of the past studies had also mainly concentrated on organizational determinants including organizational characteristics (e.g., managerial support, availability of resources and time, rewards and reinforcement, and work discretion) (Ahmed et al., 2018), knowledge management enabler (e.g., technology, structure, and culture) (Ibidunni et al., 2017; Mulhim, 2017; Rathi and Given, 2017; Adam et al., 2018; Usai et al., 2018; Zaim et al., 2019; Ode and Ayavoo, 2020).

Roots of entrepreneurship are found in the fields of economics, sociology, psychology, and anthropology (Frese and Gielnik, 2014). Entrepreneur provides opportunities by establishing new entities as well as by expanding the scope of existing organizations. Hence, entrepreneurship generates potentially new jobs and participates in the economic expansion (Branstetter et al., 2014). However, psychological elements are significant variables that may increase the impact of organizational characteristics on entrepreneurial orientation (EO). Examination of association among organizational characteristics and entrepreneurial orientation by incorporating psychological factors as moderators are significant, concerning theory as well as practice in response to a demonstration of various stages of entrepreneurial orientation by entrepreneurs in diverse organizational and psychological settings. Organizations may get various benefits from the level of entrepreneurial orientation.

Organizational determinants had shaped the outlooks of managers and employees in implementing entrepreneurial undertakings. Hence, the link between organizational factors and individual factors (e.g., innovativeness, pro-activeness, and risk-taking) may offer new insights on how the organizational factors can lead to the formation of entrepreneurial orientation and encourage employee engagement in entrepreneurial undertakings (Baskaran, 2017; Lages et al., 2017; Evansluong et al., 2019). A study on employees’ entrepreneurial orientation in the context of Pakistani manufacturing companies found that the industry faces great challenges in achieving sustainability and long-run success as a result of tough competition from neighboring countries (Mufti et al., 2020). Such challenges emerging from other countries in terms of cost advantages, capacity expansion, and increased competition in sustaining market shares have forced manufacturing companies to establish an organizational milieu that encourages employees’ entrepreneurial orientation (Baskaran, 2017). Due to such challenges, an immediate need to better understand how manufacturing companies can establish and drive more entrepreneurial-based workforce strategies has emerged. Nevertheless, since manufacturing companies in Pakistan are rather distinct in their style of operation as well as organizational practices and culture, this current study may contribute certain new knowledge and implications.

To tackle the aforementioned issues, this study had selected the resource-based view theory as the underpinning theory, considering its proven reliability and validity by numerous studies (Kesavan, 2018; Garba et al., 2019; Lee et al., 2019; Walter et al., 2019; Levasseur, 2020). Some researchers had used the resource-based view theory to examine the construct variables at an organizational level while others at the individual level (Baskaran, 2017; Evansluong et al., 2019). This current study uses the resource-based view theory to examine its implications on the entrepreneurial orientation of employees. This study aims to address the existing research gaps and expand the investigation on employees’ entrepreneurial orientation by (a) examining all levels of entrepreneurial orientation mechanisms that had not been fully explored in the context of manufacturing companies, and of which can offer valuable theoretical and practical contributions in advancing the understanding of factors that drive employees’ entrepreneurial orientation and activity (Montiel, 2018), (b) investigating the dependent and independent variables (Saleh et al., 2018), and (c) positioning these mechanisms in the context of manufacturing companies, which highly require employees’ entrepreneurial orientations (Baskaran, 2017; Evansluong et al., 2019).

## Literature review

### Entrepreneurial orientation

Entrepreneurial orientation originates from the strategy-making process (Rosim et al., 2019). Strategy making at the organizational level incorporates the aspects of planning, analysis, decision-making as well as organizational knowledge, benefits structure, and mission. In line with Tirmizi et al. (2018) who asserted the importance of strategy making in describing entrepreneurial activities, dividing resources, or setting the relevant criteria, entrepreneurial orientation delineates the contributing policies and practices that form entrepreneurial choices and activities (Sahasranamam and Sud, 2016). Additionally, the past two decades had witnessed the rise of entrepreneurial orientation (Locke and Baum, 2014; Gupta and Dutta, 2018) at the back of the substantial attention given to the aspect of entrepreneurial mindset by the researchers. Kraus et al. (2018) asserted that it denotes the organization’s emphasis on the identity and exploration of market opportunities (Hartanto et al., 2017). Ratang and Blesia (2019) asserted that a company’s orientation is based on its philosophy. Hossain and Asheq (2019) delineated entrepreneurship and entrepreneurial orientation, defining the former as a new entry and the latter as to how the new entry is initiated. Meanwhile, Kashyap et al. (2017) defined entrepreneurial orientation as the way a company manages and organizes itself while identifying weaknesses in the marketplace. Ali et al. (2017) delineated entrepreneurial orientation as the top management’s initiative in undertaking risky activities as well as being proactive and innovative. Other contemporary researchers demarcated entrepreneurial orientation as the strategic orientation in accepting distinct entrepreneurial outlooks involving practices, methods, and decision-making processes (Alhnaity et al., 2016; Capatina and Rancati, 2017; Capatina et al., 2017; Covin and Wales, 2019; Karimi and Nabavi Chashmi, 2019).

Others agree that entrepreneurial orientation plays a role in determining organizational behavior and understanding, emphasizing the proactive procurement of entrepreneurial prospects and innovative creations (Kesavan, 2018; Kraus et al., 2018; Ocak and Ozturk, 2018). Entrepreneurial orientation also allows for the reconsideration of internal and external capabilities in dealing with changing landscapes. Gupta and Dutta (2018) and Covin and Wales (2019) expanded the earlier definitions of entrepreneurial orientation by stating that despite having the same dimensions as stated by Cho and Lee (2018) such as innovativeness, pro-activeness, and risk-taking, entrepreneurial orientation also affects an organization’s processes, structures, and behaviors as demonstrated *via* its products and process innovations (Rochdi et al., 2017). The processes, practices, philosophical methods, styles, and decision-making activities of an organization, in turn, facilitate its entrepreneurial activities (Ali et al., 2017; Arham et al., 2017; Hartanto et al., 2017; Arshad and Rasli, 2018; Morgan and Anokhin, 2020). Additionally, to take advantage of the prevailing competitive environment, the organization may identify and launch corporate ventures while embracing an entrepreneurial mindset (Hossain and Asheq, 2019).

### Organizational characteristics

The success of an organization is primarily determined by its organizational characteristics (Oyewobi et al., 2016). Sahu et al. (2018) asserted that loyal employees can lead to improved organizational productivity. Hence, the top management should establish a solid management–employee relationship *via* effective communications as well as provide the necessary knowledge that would enable the employees to perform well (Giampaoli et al., 2017). Organizations should emphasize work performance as it can drive the creation of a positive working environment. Organizational goals, missions, and visions should also be clearly defined (Pook et al., 2017) while poor performances are addressed and corrected. The lack of a proper structure can negatively affect an organization’s overall performance. A well-performing organization can easily persuade its employees to follow managerial instructions, providing that they understand the reasons for those instructions. Nevertheless, to achieve organizational goals, organizational characteristics must be supported by other factors including entrepreneurial orientation.

In line with Ahmed et al. (2018), it was found that certain organizational characteristics can either drive or hinder organizational activities toward achieving organizational goals (Ahmed et al., 2018). It was also found that entrepreneurial activities are often influenced by internal organizational factors (Calisto and Sarkar, 2017). Many studies have examined the effect of internal organizational characteristics in driving employees’ entrepreneurial orientation past (Lages et al., 2017; Urban, 2017). Past studies have also investigated numerous variables such as key internal factors that drive entrepreneurial efforts. These include incentives and control systems (Kartika, 2017), organizational cultures (Kraśnicka et al., 2018; Sánchez et al., 2019), organizational structures (Hernández et al., 2017; McGee and Peterson, 2019), and managerial support (Riviezzo, 2017; Elia and Margherita, 2018). In general, entrepreneurship literature has delineated four organizational characteristics that affect entrepreneurial efforts, namely, management support, resource and time availability, work discretion, and reward/reinforcement. The next sub-sections will discuss each dimension.

### Knowledge management

This is a determining factor of knowledge management activities, which include the summarizing and sharing of knowledge resources among the employees (Saleh et al., 2018; Jennex, 2019; Singh et al., 2019). In a competitive business milieu or amid a new business phenomenon, organizations often look for new management techniques to guide their business operations (Saleh et al., 2018; Than, 2018). Most organizations believe that knowledge management can help in managing such situations (Abdulmuhsin and Tarhini, 2021). Barley et al. (2018) asserted that in creating and developing new insights and capabilities, an organization should focus on enabling communications and exchanging knowledge *via* the effective employment of knowledge management (Saleh et al., 2018). However, Bleoju and Capatina (2015) highlighted that many organizations failed to employ knowledge management effectively. Some organizations even tend to use information technology as a way of managing knowledge (Saleh et al., 2018; Abubakar et al., 2019; Singh et al., 2019). The underlying issue here entails the failure of understanding the enablers of knowledge management, which would otherwise improve the capability of employees in making good business decisions and initiating the needed actions. Jennex (2019) delineated knowledge management enablers as the factors that influence and facilitate knowledge management activities including the codification and sharing of knowledge among the employees. This is in line with the suggestion of Moghavvemi et al. (2017), who showed that apart from technological factors, individual and organizational factors are also the key enablers of knowledge sharing. Past studies had attempted to investigate the numerous knowledge management enablers that facilitate information management and acquirement, which would in turn help employees to behave entrepreneurially at the operational level (Saleh et al., 2018; Ahmad Sabir et al., 2019).

### Psychological factors as moderator

The literature highlights that psychological factors are significant that influence entrepreneurial orientation (EO) (Gump et al., 2017). Accordingly, Churchill and Bygrave (1989) and Ferreira et al. (2012) suggested that propensity to take the risk, locus of control, and aptitude of dealing with uncertainty are some important characteristics of psychological entrepreneurship. Further, Robinson et al. (1991) included self-confidence and locus of control as the key elements of psychological entrepreneurship. However, in the literature regarding entrepreneurship, the tendency of risk-taking, locus of control, the ability to deal with uncertainty, and self-confidence are highlighted variables that attained wider attention of the researchers. The tendency of risk-taking is the personal capability of a person while deciding on the situation of uncertainty (Koh, 1996). The risk-taking element notably differentiates among managers and entrepreneurs. According to Anwar and Saleem (2019), risk measurement and risk-taking are the key functions involved in entrepreneurship. Koh (1996) concluded that entrepreneurs take risks in their controlled environment and where the probability of earning profit exists. Similarly, Oosterbeek et al. (2010) stated that risk-taking tendency is the personal capability of a person while deciding on the situation of uncertainty. Shareholders and executives take the risk for attaining a competitive edge (Hoskisson et al., 2017). New opportunities are better availed by the individuals who take more risk (Bello et al., 2016).

Adeyemi-Bello (2001) described locus of control as the thoughts of a person developed by the various events that happened in his/her life. Ullah et al. (2012) defined locus of control as the faith of an individual regarding the things guided by behaviorism that include internal personal decisions and struggles, luck and fate, and other external conditions. Individuals with an internal locus of control more appropriately deal with the events that have already been happened in their past. On the other hand, individuals with an external locus of control refer most of the happening to the external forces likewise fate, luck, or other powers that impact their life performance (Koh, 1996). Accordingly, entrepreneurs have an internal locus of control as they always explore and avail new opportunities, go with innovative decisions, and have the capability of managing events in more appropriate manners (Thomas and Mueller, 2000). Individuals with an internal locus of control attentively make more struggles for success in their dealings in comparison with individuals with an external locus of control (Rotter, 1966). Various researchers discussed internal locus of control as an important entrepreneurial characteristic (Kundu and Rani, 2016). Employees and shareholders in business having the ability of inner locus of control are considered to have appropriate control over their decisions and life events. These persons are witnessed more successful because they remain active in their personal and business dealing. In the literature, various studies including Ullah et al. (2012) concluded a significantly positive association of inner locus of control with EO. Koh (1996) pointed out that a person with a higher ability of tolerance while facing a situation of uncertainty remains more successful to overcome the challenging situation.

In addition, Teoh and Foo (1997) in their research concluded that entrepreneurs are likely to have more ability of tolerance in an uncertain situation. Accordingly, entrepreneurs deal more confidently with the situation of uncertainty concerning the others who have a low level of tolerance in the uncertain situation because they lose confidence in uncertain circumstances and try to remain aside from the situation of ambiguity (Busenitz and Barney, 1997). Mangers having entrepreneurial skills are considered to exhibit more confidence and tolerance while uncertain as compared to traditional managers. On the other hand, entrepreneurs often work in the less developed structures where they have to manage the uncertain circumstances (Entrialgo et al., 2000) that ultimately impose decision-making responsibility on them.

### Resource-based view

The main part of the theory lies in the allocation of resources effectively to create innovative services for the organization for its competitive advantage. So, knowledge is needed to be managed by the organization to create value (Abualoush et al., 2018). Currently, management of knowledge in the company is considered as a prerequisite for innovation, so the company needs to create and transfer knowledge into innovation (Seidler-de Alwis and Hartmann, 2008; Martínez-Martínez et al., 2019). Knowledge combination helps the organization to reorganize its capabilities and resources to create innovation that would diffuse in the market (Martínez-Martínez et al., 2019; Raoof et al., 2021).

Knowledge management in any organization has a vital role in achieving competitive advantage and is considered to be an essential factor for organizational success where knowledge is needed for the creation of innovation (Sarala et al., 2016; Abualoush et al., 2018; Abdulmuhsin et al., 2021). To attain a competitive advantage, organizations need to combine resources and competencies (Ferraris et al., 2019). Knowledge management is practiced to upgrade the effectiveness in sustaining and creating the intellectual assets of an organization (Ramadan et al., 2017). KM capabilities consist of KM enablers and KM processes (Iqbal et al., 2019). According to the study of Torres et al. (2018), KM processes include acquisition, creation, and sharing of knowledge to retain competitive advantage (Basheer M. et al., 2019).

### Concept of entrepreneurial orientation

Entrepreneurial orientation has been considered as a predictor of KM processes such as knowledge utilization (Wach et al., 2018), creation of knowledge (Li et al., 2019), and sharing knowledge (Latif et al., 2020). Additionally, EO is a strategic part of the company, which is linked with the development of policies and procedures for entrepreneurial actions for the attainment of competitive advantage (Martens et al., 2018). Anderson et al. (2015) suggested that EO is the strategic position of the company related to entrepreneurial development of knowledge management that is beneficial in determining new opportunities for the business. Conceptually, EO is the combination of risk-taking, proactive behaviors, and innovations in the firm (Li et al., 2019).

Martens et al. (2018) argued that entrepreneurship is all about risk-taking and is associated with fundamental policies and practices for the development of entrepreneurial actions that leads to competitive advantage. To create a competitive advantage, EO is conceptually operated by decision-makers (de Guimaraes et al., 2018). These policies and practices are described as the key mechanisms for the knowledge integration of individuals (Grant, 1996; Nuseir et al., 2020). It is proposed that EO has a significant impact on KM processes (Gupta and Moesel, 2007). Stuetzer et al. (2018) also supported this conclusion and found that initiatives of management such as experimentation, key constituents of EO, and risk-taking effect the creation and sharing of knowledge.

## Hypothesis development

Employees’ entrepreneurial orientation has undergone a conceptual evolution in the past two decades focusing on the pursuit of new prospects, risk-taking, and innovativeness (Hernández et al., 2017; Ahmed et al., 2018; Cho and Lee, 2018; Gupta and Dutta, 2018; Hameed et al., 2018; Covin and Wales, 2019; Hossain and Asheq, 2019). This section discusses the hypotheses developed in this study. There are three variables of which mutual relationships are examined in this study, namely: (i) organizational characteristics, (ii) knowledge management enablers, and (iii) employees’ entrepreneurial orientation. This section explains the development and description of the hypothesis for each variable. Organizational characteristics are defined as an organization’s endeavor in facilitating and promoting entrepreneurial behavior and activities through the provision of necessary resources. Organizational characteristics are said to affect employees’ entrepreneurial orientation. There are various factors why organizational discretion drives employees’ entrepreneurial orientation (Mugabira, 2017) including interest improvement and self-esteem (Hobbs et al., 2020). The entrepreneurial mindset is created with the prevalence of employee agreement, effective cooperation, creativity, and shared responsibility (Lee et al., 2017), all of which help improve employee engagement, responsibility, and awareness of entrepreneurial efforts. This also includes how the top management drives the entrepreneurial orientation mentality within the organization which will, in turn, affect employees’ entrepreneurial behavior (Han and Park, 2017), the shared visions for the future, the acknowledgment and approval of new ideas, the provision of needed resources to initiate entrepreneurial orientation, and the successful introduction and development of products. Ahlstrom et al. (2018) studied the effects of organizational characteristics on entrepreneurial orientation and found a positive relationship between the two variables. Despite the abundance of studies on organizational characteristics, very few had examined the effects of organizational characteristics on employees’ entrepreneurial orientation particularly in the context of the manufacturing industry. This current study thus proposes the hypothesis below.

Management support has been highlighted in many past studies (Hwang and Suh, 2017; Kim, 2019; Lee and Lee, 2020). Managers have greater knowledge of the supply chain as they are the ones responsible for their organization’s strategic plans to remain competitive in the marketplace (Prajogo et al., 2016). Hence, the top management should nurture its relationship with employees by engaging its time as well as the organization’s personnel and financial resources (Abu et al., 2019). The resource-based view theory also proposes the important role of management support within organizations. The allocation of attention to certain activities explains why some organizations can successfully nurture employee engagement. Employee engagement can be improved by directing the employees’ energy and effort on certain activities. Hence, organizational intervention is needed to yield positive outcomes from entrepreneurial activities. The top management’s expectations of the outcomes of entrepreneurial activities are a key component in this context. Managerial support can empower employees’ entrepreneurial orientation. The studies by Arshi and Burns (2018) and Khoshmaram et al. (2020) also corroborated the significance of management support in facilitating the achievement of organizational goals as well as in encouraging and advancing entrepreneurial activities in organizations. According to Bien and Arena (2018) and Deken et al. (2018), the top management should take the responsibility of fulfilling organizational goals to achieve organizational success. Meanwhile, Soomro and Honglin (2018) and Nehles and Veenendaal (2019) asserted that management support is one of the key quality measurements of the entrepreneurial behavior of employees. Thus, the following hypothesis has planned for the construct:

H_1_: *Management support has a significant positive impact on employees’ entrepreneurial orientation*.

H_2_: *Management support has a significant positive impact on knowledge process capabilities*.

Baskaran (2017) stated that time and resource availability can empower entrepreneurial efforts (Kartika, 2017; Kuratko and Hoskinson, 2018). Employees who are provided with adequate time and resources are better empowered in carrying out entrepreneurial activities. The resources in this context include cash and time. This aspect has been a continuous focus in the most entrepreneurship literature. There is a crucial need to ensure the availability of assets and the capability of existing mechanisms, frameworks, and procedures in the effort to nurture entrepreneurial behavior among the employees. This includes assessing the present workload of employees and ensuring that they have adequate time to complete it. This suggestion is in line with the findings of Franz (2019), which demonstrated the need for innovation in ensuring the availability of resources. Additionally, the organizational structure should take the long- and short-term organizational goals into consideration, giving the employees adequate time to complete their daily tasks (Raghuvanshi et al., 2017; Evansluong et al., 2019). Thus, the following hypothesis has been planned for the construct:

H_3_: *Resource and time availability has a significant positive impact on employees’ entrepreneurial orientation.*

H_4_: *Resource and time availability has a significant positive impact on knowledge process capabilities.*

This is a vital aspect in ensuring employee engagement, creating efficient employee behavior, and retaining employee loyalty and commitment (Awada et al., 2019; Ullah et al., 2020). The term “total reward” refers to monetary and non-monetary, direct and indirect, as well as elemental and extrinsic rewards or reinforcements that are expected to improve employee well-being, satisfaction, and productivity (Van Rooyen, 2018). Employees will not demonstrate entrepreneurial behavior if they do not perceive any benefits of doing so. Employees expect recognition for their substantial contribution or excellent work performance. Hasbi et al. (2020) asserted that employees are more likely to remain loyal and contribute more to their employing organization when they are rewarded accordingly for their work and demonstrated that employees are more willing to accomplish challenging tasks when their employment terms incorporate rewards and reinforcements, opportunities for career advancement, and recognition schemes for their critical contributions. Baskaran (2017) asserted the importance of rewards and reinforcements in encouraging employee engagement, despite the suggestions that the top management plays a bigger role in driving entrepreneurial objectives. The role of knowledge in encouraging engagement has also been extensively studied and proven (Santoro et al., 2018). According to Haq and Faridi (2020), knowledge is a valuable organizational asset. Prouska et al. (2016) found that rewards given by the top management could help to improve the performance of weaker employees as well as the work environment as a whole, thus positively affecting business performance. Thus, the following hypothesis has been planned for the construct:

H_5_: *Reward has a significant positive impact on employees’ knowledge process capabilities.*

H_6_: *Reward has a significant positive impact on employees’ entrepreneurial orientation.*

Past studies had identified two terms that mean the same, i.e., occupation independence and employee work discretion. They are also sometimes referred to as employment control and choice scope. Raghuvanshi et al. (2017) defined work discretion as an organization’s commitment toward decision-making and the prospects of carrying out entrepreneurial endeavors while being able to manage the setbacks that come with those endeavors. The top management should be able to tackle any setbacks accompanying their entrepreneurial activities (Baskaran, 2017; Evansluong et al., 2019). Additionally, they should also allow an adequate level of decision-making for the employees, complete with adequate oversight as demonstrated by the work discretion measurement. In line with the suggestions of past studies, Gawke et al. (2019) pointed out that employees should not be reprimanded for making mistakes and that they should be included in entrepreneurial endeavors. Thus, the following hypothesis has been planned for the construct:

H_7_: *Work discretion has a significant positive impact on employees’ entrepreneurial orientation.*

H_8_: *Work discretion has a significant positive impact on knowledge process capabilities.*

Numerous studies in the past two decades have investigated various knowledge management process capabilities (Ibidunni et al., 2017; Mulhim, 2017; Rathi and Given, 2017; Adam et al., 2018; Usai et al., 2018; Zaim et al., 2019; Ode and Ayavoo, 2020). The next section discusses the application of knowledge management process capabilities. Earlier studies such as that of Santoro et al. (2018) and Antunes and Pinheiro (2020) asserted that tacit and explicit knowledge should derive even more significant outcomes. Kazempourian et al. (2020) studied this proposition in the context of Australia by utilizing the case study approach in exploring the transformation of tacit knowledge into explicit knowledge on the top of investigating three knowledge management enablers, namely, culture, organizational structure, and technologies (Singh et al., 2019). The study demonstrated that organizations perceive culture and organizational structure as significant in transforming tacit knowledge into explicit knowledge. Nevertheless, incentives or exchange mechanisms are needed in sharing the knowledge embedded in the employees’ minds (Singh et al., 2019). Yework (2020) examined the effect of organizational factors on knowledge transfer in the context of the public sector. In the context of Vietnamese IT companies, Owusu et al. (2018) found that communal culture, communication systems, transformative influence, and knowledge automation serve as crucial knowledge management enablers that influence knowledge sharing.

Sayyadi (2020) examined the effect of knowledge management enablers on knowledge management processes using the research model developed by Lee and Choi. They found that the variables of technology and culture affect knowledge management processes while the variable of structure does not. Elezi and Bamber (2018) examined the ranking and weight of knowledge management in the context of university academic staff and students. They found that organizational culture demonstrates the greatest importance while organizational structure the least. As previously mentioned, many of the knowledge management studied in the past are overlapped (Rathi and Given, 2017; Adam et al., 2018; Elezi and Bamber, 2018; Singh et al., 2019). A comparative analysis of these past studies showed that there are no common or generic sets of knowledge management enablers. Yet, knowledge management enablers need to be cohesive (Prado et al., 2020). Yasir and Majid (2017) indicated that knowledge management enablers should be observed from a social-technical standpoint. While the factors of the employee, relationships, and organizational structure denote the social opinion, the technical standpoint deals with the technological requirements in converting inputs into outputs (Brocke et al., 2018). Hence, based on the current study’s objective, knowledge process capabilities are deemed as knowledge management enablers in an organization. The next analysis focuses on the capabilities needed to drive employee knowledge management and entrepreneurial behavior.

The topic of knowledge management capabilities has been extensively studied in the field of knowledge management (Kane, 2017). From the standpoint of infrastructure, Zaim et al. (2019) found that knowledge process capabilities act as knowledge management enablers. Knowledge is also indicated to play a vital role in establishing entrepreneurial orientation (Nallaluthan et al., 2020). Additionally, sufficient knowledge is needed in determining the cause and effects of engaging employees in entrepreneurial endeavors. Hence, this current study proposes the following hypothesis:

H_9_: *Knowledge process capabilities have a significant positive impact on employees’ entrepreneurial orientation.*

In terms of the significance of internal and external information, Usai et al. (2018) pointed out that technology facilitates an organization in identifying the source of information derived from internal and external environments. An organization can achieve its short-term and long-term objectives if its employees are adequately equipped with the proper knowledge on the top of having knowledge management enablers in place. The adoption of technology alone does not guarantee organizational success; rather, the technology must also be user-friendly to enable the promotion of the organization’s system and its usage among the employees, so that decisions are made based on sufficient information. Additionally, the needs of employees must be addressed as a part of the technological application development to enhance the benefits of the technological investment, to ensure that the technology’s intended purpose is attained, and to foster greater entrepreneurial decision-making among the employees. Some studies had investigated the relationship between knowledge management and entrepreneurial orientation (Kashyap et al., 2017; Adam et al., 2018; Hossain and Asheq, 2019; Nallaluthan et al., 2020). Adam et al. (2018) found a positive relationship between the two variables in the context of small and medium enterprises (SME) performance. This indicates that knowledge does affect employees’ entrepreneurial orientation. However, despite the abundance of studies on knowledge management, very few had examined the relationship between knowledge management enablers and employees’ entrepreneurial orientation (Ha et al., 2016). Hence, this current study proposes the following hypothesis:

H_10_: *Knowledge process capabilities mediates the relationship between the resource and time availability and employees’ entrepreneurial orientation.*

H_11_: *Knowledge process capabilities mediates the relationship between the rewards and employees’ entrepreneurial orientation.*

H_12_: *Knowledge process capabilities mediates the relationship between work discretion and employees’ entrepreneurial orientation.*

H_13_: *Knowledge process capabilities mediates the relationship between the management support and employees’ entrepreneurial orientation.*

Expectancy theory is linked with cognitive processes, in the given situation entrepreneur combine their needs and expectations in terms of organizational characteristics. This theory explains why a person chooses to be an entrepreneur. Additionally, expectancy theory consists of two parts effort-performance link, which elaborates the inputs of an employee in the organization and the performance according to the facilities and characteristics of the organization. Hence, the variables organizational characteristics and employee’s entrepreneurship is linked while psychological factors may moderate the relationship between organizational characteristics and employee’s entrepreneurship. In the literature, studies demonstrate that psychological factors can increase the impact of organizational factors concerning the study of Palmer and Weiss (2021). So, Churchill and Bygrave (1989) recommended propensity of risk-taking, internal locus of control, and facing uncertain situations as significant factors of psychological entrepreneurship. Furthermore, Robinson et al. (1991) included self-confidence and locus of control as the key elements in psychological entrepreneurship. While most widely used elements of entrepreneurship are the tendency of risk-taking, locus of control, the ability to deal with uncertainty, and self-confidence, risk-taking propensity is the personal capability of a person while deciding on an uncertain situation (Koh, 1996). Notably, risk-taking ability distinguishes entrepreneurs from managers (1983). Similarly, Oosterbeek et al. (2010) defined propensity in similar meanings. Executives and members take a risk for attaining competitive advantage (Hoskisson et al., 2017). Higher risk-taking individuals avail themselves of a maximum of new opportunities (Bello et al., 2016). Locus of control is described by Rotter (1966) as a person’s thoughts formed by the various events that happened in his/her life. Ullah et al. (2012) explain the locus of control in the context of behaviorism by incorporating internal personal decisions, luck and fate, and other external forces. Individuals having an internal locus of control more properly deal with the events that they had experienced in past. However, individuals with an external locus of control blame external forces often such as fate, luck, or other powers that influence performance (Koh, 1996; Zafar et al., 2021). Accordingly, entrepreneurs always explore and avail new opportunities because of internal locus of control, go with innovative decisions, and have better capability of managing events (Thomas and Mueller, 2000).

H_14_: *Propensity to take risks moderates the relationship of resource and time availability with employees’ entrepreneurial orientation.*

H_15_: *Propensity to take risks moderates the relationship of rewards with employees’ entrepreneurial orientation.*

H_16_: *Propensity to take risks moderates the relationship of work discretion with employees’ entrepreneurial orientation.*

H_17_: *Propensity to take risk moderates the relationship of management support with employees’ entrepreneurial orientation.*

H_18_: *Locus of control moderates the relationship of resource and time availability with employees’ entrepreneurial orientation.*

H_19_: *Locus of control moderates the relationship of rewards with employees’ entrepreneurial orientation.*

H_20_: *Locus of control moderates the relationship of work discretion with employees’ entrepreneurial orientation.*

H_21_: *Locus of control moderates the relationship of management support with employees’ entrepreneurial orientation.*

## Materials and methods

As many as 438 questionnaires were dispersed to the participants in the selected university. In an attempt to obtain a higher response rate, the questionnaires were distributed manually (by hand) by the researcher to ascertain data reliability and validity (Kante et al., 2018). The attempt yielded 334 reverted questionnaires, i.e., a response rate of 76.2%, which fulfills the recommended threshold by Islam (2019). Out of the 374 returned questionnaires, 43 were unusable as some of the essential sections in the questionnaires were left incomplete. The remaining 291 questionnaires were suitable to be used in the analysis. This accounts for 66.4% of the usable response rate, which is sufficient for analysis as it exceeds the 30% minimum response rate as suggested by Ringle et al. (2018) (refer to Table 1).

**TABLE 1:** Response rate.

Response	Frequency	Percentage
No. of distributed questionnaires	438	100
Returned questionnaire	334	76.2
Excluded questionnaire	43	9.8
Returned and usable questionnaire	291	66.4
Sample after data screening	274	62.5

Based on the observed variables (items), the chi-square threshold is suggested at 144.12 at (*p* = 0.001). Any Mahala Nobis values that go beyond this threshold are omitted. Based on this criterion, 17 of the cases were found to be multivariate outliers, namely, 1, 3, 41, 84, 87, 88, 92, 96, 97 101, 102, 104, 112, 137, 156, 255, and 259 and hence omitted from the dataset as these outliers can affect the estimation of the result. Conclusively, after the deletion of the 17 outliers, the dataset was left with 274 for the analysis of the measurement and structural models. Table 2 presents the respondents’ demographics. Almost two-thirds of the total respondents were 36 years old or above. Approximately 77.5% of them have been working for 11 years or more in the manufacturing industry. About 82.0% hold a master’s degree.

**TABLE 2:** Demographic profile.

**Age**		
20–27	11	4.01
28–35	68	24.81
36–43	77	28.10
44–50	54	19.70
Above 50	64	23.3
**Experience**		
Below 3 years	12	4.40
3–7 years	21	7.70
7–11 years	33	12.08
11–15 years	43	15.75
Above 15 years	164	60.07
**Qualification**		
Bachelor	47	17.15
Master	225	82.11
PhD	02	1.00

## Analysis and results

Henseler et al. (2015) in their seminal study argued goodness-of-fit (G-O-F) as an inappropriate technique for the model estimation, which was further confirmed by Hair et al. (2019). In their study, they broached this argument based on the inference that the index of goodness-of-fit (G-O-F) as using PLS is unable to distinguish between the valid and invalid models with simulated data. Thereby following the Henseler et al. (2015), the study has used a two-step process for assessing and reporting the results. The process consists of (1) assessment of the measurement model and (2) assessment of the structural model (Hair et al., 2019).

**FIGURE 1:**
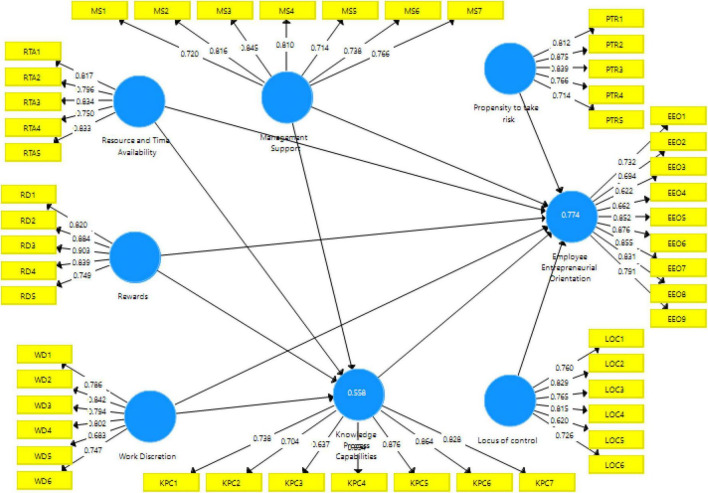
Measurement model.

The measurement model (shown in Figure 1) was assessed by determining the reliability, internal consistency reliability (ICR), and content validity of the individual items, which include the convergent validity and discriminant validity (Hair et al., 2016).

The outer loading of each construct is used to measure the individual item reliability and following Hair et al. (2019), the items with loadings 0.70 or above are omitted from the final analysis. The results are reported in Table 3 below.

**TABLE 3:** Outer loadings.

Construct	Indicators	Loadings		Alpha	CR	AVE
Employees’ entrepreneurial orientation	EEO1	0.732		0.914	0.930	0.598
	EEO2	0.694				
	EEO3	0.622				
	EEO4	0.662				
	EEO5	0.852				
	EEO6	0.876				
	EEO7	0.855				
	EEO8	0.831				
	EEO9	0.791				
Knowledge process capabilities	KPC1	0.738	0.877		0.907	0.589
	KPC2	0.704				
	KPC3	0.637				
	KPC4	0.854				
	KPC5	0.876				
	KPC6	0.864				
	KPC6	0.828				
Management support	MS1	0.720	0.888		0.891	0.599
	MS2	0.816				
	MS3	0.845				
	MS4	0.810				
	MS5	0.714				
	MS6	0.738				
	MS7	0.766				
Resource and time availability	RTA1	0.817	0.865		0.903	0.651
	RTA2	0.796				
	RTA3	0.834				
	RTA4	0.750				
	RTA5	0.833				
Rewards	RD1	0.820	0.895		0.923	0.707
	RD2	0.884				
	RD3	0.903				
	RD4	0.839				
	RD5	0.749				
Work discretion	WD1	0.786	0.871		0.901	0.604
	WD2	0.742				
	WD3	0.794				
	WD4	0.802				
	WD5	0.683				
	WD6	0.747				
Propensity to take risk	PTR1	0.812	0.792		0.859	0.567
	PTR2	0.875				
	PTR3	0.839				
	PTR4	0.766				
	PTR5	0.714				
Locus of control	LOC1	0.760	0.852		0.887	0.571
	LOC2	0.829				
	LOC3	0.865				
	LOC4	0.815				
	LOC5	0.620				
	LOC6	0.726				

To measure the latent constructs and composite reliability (CR) we have also used the standard algorithm technique. Hair et al. (2016), Henseler et al. (2016), and Akter et al. (2017) have suggested that 0.6 is the threshold value for CR while other researchers claimed that the minimum acceptable value is 0.7 (Hair et al., 2017; Shuhaiber, 2018; Shiau et al., 2019; Hameed et al., 2020) Raghuvanshi et al. (2017). To check the construct validity, it is important to calculate discriminant and convergent validity. Hair et al. (2019) also cited a value of 0.60 or higher for the composite reliability coefficient. The composite reliability coefficients for the latent constructs are also shown in Table 3. We have determined the compliance between the specific measurement tool and theoretical concepts using the convergent validity. Specifically, the convergent validity checks whether the measuring scale has characterized the corresponding attributes (Naala et al., 2017). Moreover, to measure the convergent validity, we have calculated the factor loadings, composite reliability, and average variance extracted (AVE) (Ong and Puteh, 2017). The convergent validity can only be determined only when all items are correlated for a specific construct. In this study to check the convergent validity, we have calculated the values of AVE (Hair et al., 2016, 2017; Richter et al., 2016). The results of PLS algorithm indicates that the values of all the AVE are greater than the minimum acceptable value that is 0.5, hence indicating sufficient convergent validity.

For the discriminant validity, we generally employ the most important measures that are cross-loading techniques and the Fornell–Larcker criterion, while the measurement of discriminant validity we have also checked that the irrelevant theoretical concepts are statistically irrelevant as well. We have taken the AVE square root to apply the Shuhaiber (2018) criterion, in the correlational matrix which is placed diagonally (shown in Table 4). We have also compared the values between the squared correlation and square root values of constructs. If the values of squared correlation among the constructs are less than the square root values, then the discriminant validity will be established (Hair et al., 2017). The square roots of the AVE average were found to be larger than the latent constructs’ correlations with each other, which decisively suggest that sufficient discriminant validity has been achieved (Akter et al., 2017; Naala et al., 2017; Shuhaiber, 2018).

**TABLE 4:** Fornell–Larcker criterion.

	EEO	KPC	LOC	MS	PTR	RTA	RD	WD
EEO	0.774							
KPC	0.525	0.768						
LOC	0.532	0.745	0.755					
MS	0.489	0.686	0.637	0.774				
PTR	0.622	0.657	0.657	0.554	0.753			
RTA	0.533	0.597	0.623	0.689	0.490	0.807		
RD	0.495	0.620	0.550	0.523	0.726	0.578	0.841	
WD	0.522	0.632	0.544	0.467	0.624	0.677	0.712	0.777

Overall, the findings for the measurement model show that it has no reliability and validity issues. Next, the structural model is assessed to determine the significance of the correlation between and among the variables.

**FIGURE 2:**
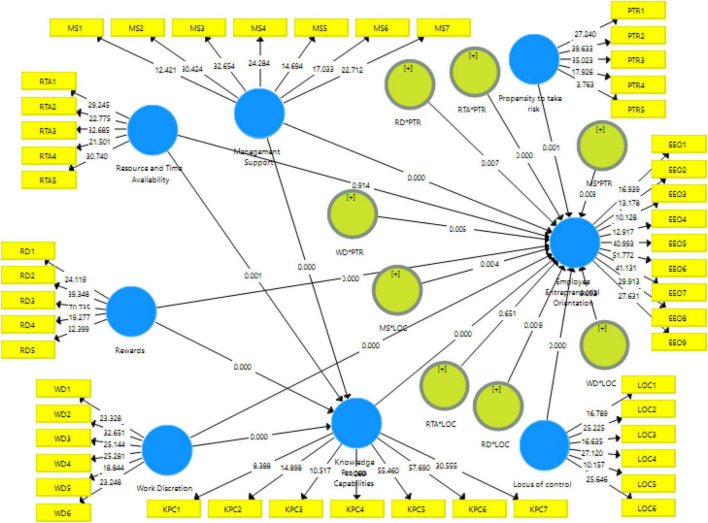
Structural model.

The relationship between the constructs of the model is measured in the structural model (Hair et al., 2017). Consequently, it helps in the determination of relationships between exogenous and endogenous constructs of the model. In the measurement of the structural model, the calculation of *R*-square, path coefficients significance, or *t*-values are important (Hair et al., 2016; Henseler et al., 2016; Akter et al., 2017). The path coefficients describe the strength of the relationship among dependent, independent, and assumed variables (Shiau et al., 2019). To test the hypothesis, significance of path coefficients, and to achieve *t*-values, we have employed the bootstrapping procedure by following the suggestions of Shuhaiber (2018). For this purpose, we have used a sample of 5,000 bootstraps. Meanwhile, there are different factors due to which the *t*-values are higher than 0, such as confidence interval, level of freedom, and the directivity of hypothesis (Hair et al., 2017). The assessment of the structural model employs the bootstrapping procedure by running 500 bootstrap samples on 274 cases to evaluate the path coefficients’ significance (Hair et al., 2016; Shuhaiber, 2018; Islam, 2019). The direct and mediation estimates of the structural model are shown in Figure 2.

For the estimation of hypotheses of the study, PLS-SEM analysis was conducted. Table 5 indicated the results of structural model analysis for the direct relationship of organizational factors with knowledge process capabilities and employees’ entrepreneurial orientation. The results show that all direct hypotheses are accepted on statistical grounds except H_3_. The results show that resource and time availability has no significant impact on employees’ entrepreneurial orientation. All other factors have a significant relationship with employees’ entrepreneurial orientation as well as knowledge process capabilities. Therefore, H_1_, H_2_, H_4_, H_5_, H_6_, H_7_, H_8_, and H_9_ are supported.

**TABLE 5:** Structural model assessment (direct effect results and decision).

Hypotheses	Relationship	Beta	STDEV	T statistics	*P*-values
H1	MS→EEO	0.542	0.093	30.796	0.000
H2	MS→KPC	0.272	0.084	16.912	0.000
H3	RTA→EEO	−0.014	0.025	0.566	0.914
H4	RTA→KPC	0.494	0.067	7.368	0.000
H5	RD→EEO	0.125	0.053	6.259	0.000
H6	RD→KPC	0.092	0.03	3.035	0.000
H7	WD→EEO	0.056	0.022	2.498	0.000
H8	WD→KPC	0.078	0.033	2.326	0.000
H9	KPC→EEO	0.108	0.033	5.456	0.000

Authors’ estimates based on survey data.

For the estimation of the mediation role of knowledge process capabilities between the relationship of organizational factors with employees’ entrepreneurial orientation, the bootstrapping procedure is adopted. The results of the analysis (shown in Table 6) indicated that knowledge process capabilities do not mediate the association of management support with employees’ entrepreneurial orientation while it mediates the relationship of resource and time availability, rewards, and work discretion with employees’ entrepreneurial orientation. Therefore, H_11_, H_12_, and H_13_ are supported.

**TABLE 6:** Structural model assessment [indirect (mediation) effect results and decision].

Hypotheses	Relationship	Beta	STDEV	T statistics	*P*-values
H10	MS→KPC→EEO	−0.069	0.041	1.665	0.097
H11	RTA→KPC→EEO	0.140	0.044	3.179	0.002
H12	RD→KPC→EEO	0.238	0.051	4.694	0.000
H13	WD→KPC→EEO	0.174	0.043	4.093	0.000

Authors’ estimates based on survey data.

Table 7 specified the results of moderation analysis. The results show that the propensity to take risks significantly moderates the relationships of organizational factors with employees’ entrepreneurial orientation. Moreover, locus of control moderates the relationship of management support, rewards, and work discretion with employees’ entrepreneurial orientation.

**TABLE 7:** Structural model assessment (moderation effect).

Hypotheses	Relationship	Beta	STDEV	T statistics	*P*-values
H14	MS*PTR→EEO	0.232	0.079	2.909	0.008
H15	RTA*PTR→EEO	0.121	0.059	2.037	0.000
H16	RD*PTR→EEO	0.189	0.066	2.880	0.007
H17	WD*PTR→EEO	0.420	0.064	6.609	0.005
H18	MS*LOC→EEO	0.233	0.067	3.504	0.004
H19	RTA*LOC→EEO	0.140	0.044	0.179	0.651
H20	RD*LOC→EEO	0.147	0.032	4.567	0.004
H21	WD*LOC→EEO	0.174	0.043	4.093	0.008

Authors’ own estimates based on survey data.

To check the output variables’ difference which occurred because of predictor variables, we have calculated the coefficient of determination of *R*-square as per the recommendations of Richter et al. (2016), Hair et al. (2017), and Naala et al. (2017). In the structural model, the estimation of *R*-square is a key criterion with a normal range between 0 and 1. Naala et al. (2017) indicated that *R*-squared values of 0.67, 0.33, and 0.19 in the PLS-SEM algorithm, respectively, denote substantial, moderate, and weak effects. The *R*-squared value for the endogenous latent variable in this context is presented in Table 8.

**TABLE 8:** R-square.

	R-square adjusted
Employees’ entrepreneurial orientation	0.774
Knowledge process capabilities	0.558

Hence, by following the criteria suggested by both Naala et al. (2017) and Ringle et al. (2018), the endogenous latent variables showed a significant level of *R*-squared.

## Conclusion

There is a lack of studies on the effect of organizational characteristics on employees’ entrepreneurial orientation in the context of Pakistan, which save for several existing ones that had attempted to examine the said relationship (Oyewobi et al., 2016; Prouska et al., 2016; Baskaran, 2017; Ahlstrom et al., 2018; Aremu et al., 2018; Ershadi et al., 2019; Evansluong et al., 2019). In terms of knowledge process capabilities, none of the small numbers of available studies had managed to establish the extent to which knowledge can influence employees’ entrepreneurial orientation in the context of manufacturing industries (Kadhim et al., 2018; Seo, 2020). Hence, this current study attempts to add to the existing body of the literature by examining this relationship in the context of manufacturing industries in Pakistan.

Based on the discussions above and on the resource-based view theory introduced by Jogaratnam (2017), this current study aims to determine the effects of organizational characteristics and knowledge process capabilities on employees’ entrepreneurial orientation. First, this study intends to investigate the relationship among organizational characteristics, knowledge process capabilities, and employees’ entrepreneurial orientation. Second, it attempts to investigate the relationship among organizational characteristics, knowledge process capabilities, and entrepreneurial orientation. Thirdly, it investigates the moderating role of psychological factors on the relationship of organizational characteristics with employees’ entrepreneurial orientation.

Organizational characteristic plays a crucial role in driving entrepreneurial orientation. Meanwhile, the various dimensions under organizational characteristics are proposed to affect employees’ entrepreneurial orientation. Many factors of organizational discretion have been identified to drive entrepreneurial orientation (Mugabira, 2017; Hobbs et al., 2020). The entrepreneurial mindset can only be developed with the prevalence of employee empowerment, enthusiastic support, creativity, and shared authority (Lee et al., 2017) as such qualities can boost employee engagement, responsibility, and awareness of entrepreneurial efforts (Basheer M. F. et al., 2019; Kafafi, 2019). The findings of previous studies revealed that organizational characteristics can substantially drive employees’ entrepreneurial orientation.

The topic of knowledge management capabilities has been extensively addressed in the knowledge management literature (Kane, 2017). Knowledge process capabilities play a vital role in determining entrepreneurial orientation (Nallaluthan et al., 2020). Several studies had examined the effect of knowledge management on entrepreneurial orientation (Kashyap et al., 2017; Adam et al., 2018; Hossain and Asheq, 2019; Nallaluthan et al., 2020). Knowledge process capabilities were identified to have a significant effect on employees’ entrepreneurial orientation (Ha et al., 2016; Kashyap et al., 2017; Adam et al., 2018; Hossain and Asheq, 2019; Nallaluthan et al., 2020). This finding proves that knowledge process capabilities can drive employees’ entrepreneurial orientation in the context of manufacturing industries. Moreover, psychological factors also play an important role in making entrepreneurial mindset and in entrepreneurial orientation. Psychological factors significantly moderate the relationship of organizational characteristics with employees’ entrepreneurial orientation.

The entrepreneurial process is thus initiated *via* the generation of innovative ideas that are attributed to various reasons. The influencer which is both the source of formulation and the coordinator for concept creation is the organization’s “existing knowledge,” which systematizes its *status quo* and leads the entrepreneur to wider horizons and new ideas. By examining existing organizational knowledge, innovative employees can identify the pluses and deficits of their organization. Organizational entrepreneurship hence entails the generation of valuable and beneficial ideas as well as the management of existing knowledge. Following the discovery of innovative ideas, corporate entrepreneurs will then seek to establish proper opportunities. This refers to the proper usage of ideas, which would otherwise go to waste if they are not applied at the right time and in the right place. Due to this, entrepreneurs are constantly on the lookout for information, whether internally or externally. Following the implementation of those ideas, testing will be carried out. Regardless of whether the innovation process is a success or failure, the learning and experiences derived from the whole process can improve the organization’s entrepreneurial endeavors. Such learning and experiences can be captured and circulated throughout the organization. This can facilitate the next batch of innovators in improving the failed process or maintaining the successful one for further usage. The initiation of entrepreneurial endeavors can occasionally result in re-innovation. This means that the knowledge derived from the innovation process if managed well can improve existing organizational knowledge.

## Implications and future works

This study has contributed many new insights on the issues related to employees’ entrepreneurial orientation in the context of manufacturing industries in Pakistan. To the best of the author’s knowledge, the current study is the first of its kind to examine the relationships among organizational characteristics, knowledge management enabler, and employees’ entrepreneurial orientation in the aforementioned context. Additionally, this study has also attempted to enrich the existing body of knowledge by investigating the mediating effect of knowledge process capabilities on employees’ entrepreneurial orientation *via* the PLS-SEM analysis. This study provides several contributions to the field of study by merging the effects of organizational characteristics, knowledge process capabilities, and entrepreneurial orientation. The next sub-sections will elaborate on the research contributions. This study also investigates the moderating role of psychological factors on the relationship of organizational characteristics with employees’ entrepreneurial orientation.

This study offers several theoretical and practical implications. Adam et al. (2018) asserted the importance of entrepreneurial orientation in ensuring the survivability of organizations in the ever-changing marketplace (Maity et al., 2020). Numerous studies have explored the effects of entrepreneurial orientation on organizational performance (Hartanto et al., 2017; Arshad and Rasli, 2018). Yet, very few had investigated the effect of employees’ entrepreneurial orientation in the context of the manufacturing sector (Baskaran, 2017; Evansluong et al., 2019). Hence, this study had added to the understanding of the relationship among organizational characteristics, knowledge process capabilities, and employees’ entrepreneurial orientation. The current research framework was developed based on the findings of past studies and is used to test the established hypotheses.

This study contributes to the existing body of the literature by emphasizing the importance of organizational characteristics in the context of the Pakistani manufacturing sector. This contribution extends to the investigation of ambiguities in the relationship between organizational characteristics and employees’ entrepreneurial orientation as there are very few studies that had examined the said relationship particularly in the context of the Pakistani manufacturing industry (Henzab et al., 2018). Second, this study highlights the most significant knowledge process capabilities that drive employees’ entrepreneurial orientation in line with the findings of past studies. Hence, this study also contributes to the existing body of management literature by examining the effects of knowledge process capabilities on employees’ entrepreneurial orientation (Adam et al., 2018; Nallaluthan et al., 2020). Due to the literature-supported findings, both academicians and practitioners have agreed on the importance of knowledge management enabler strategies in developing employees’ entrepreneurial orientation; in short, this study confirms the significant influence of knowledge management enablers in establishing employees’ entrepreneurial orientation (Adam et al., 2018; Nallaluthan et al., 2020).

## Data availability statement

The original contributions presented in this study are included in the article/supplementary material, further inquiries can be directed to the corresponding author.

## Author contributions

All authors contributed equally to the conception and design of the study and approved the submitted version.
